# Potential mechanisms of metabolic reprogramming induced by ischemia–reperfusion injury in diabetic myocardium

**DOI:** 10.1111/1753-0407.70018

**Published:** 2024-10-25

**Authors:** Haping Ma, Jiyao Zhao, Yan Zheng, Junjie Wang, Yultuz Anwar, Yuxuan He, Jiang Wang

**Affiliations:** ^1^ The First Affiliated Hospital of Xinjiang Medical University Xinjiang Uygur Autonomous Region Ürümqi China

**Keywords:** diabetic myocardium, energy metabolism, ischemia–reperfusion injury, metabolic reprogramming, vulnerability

## Abstract

**Objective:**

This study aimed to explore metabolic reprogramming in diabetic myocardium subjected to ischemia–reperfusion injury (I/RI) and potential mechanisms.

**Background:**

Increased vulnerability after I/RI in diabetic myocardium is a major cause of the high prevalence of perioperative adverse cardiac events, and the specific alterations in energy metabolism after I/RI in diabetic myocardium and the impact on increased vulnerability are not fully understood.

**Methods:**

Metabolomic methods were used to explore the differences and characteristics of metabolites in the heart tissues of four groups, and then, single‐cell RNA sequencing (ScRNA‐seq) was used to explore the potential mechanism of metabolic reprogramming.

**Results:**

It was found that the fatty acid metabolism of db/db mouse I/RI (DMI) showed a significant upward trend, especially the metabolites of ultra‐long and medium‐long‐chain fatty acids; the metabolic flow analysis found that the U‐13C glucose M + 6 was significantly higher in the C57BL mouse sham operation (NM) group than in the db/db mouse sham operation (DM) group, and in the C57BL mouse I/RI (NMI) than in the DMI group. Compared with the NMI group, the intermediate metabolites of glycolysis and tricarboxylic acid (TCA) cycle were significantly reduced in the DMI group; all comparisons were statistically significant (*p* < 0.05), indicating that the glucose uptake of diabetic myocardetis, the ability of glucose glycolysis after I/RI, and the contribution of glucose to TCA were significantly reduced. The results of ScRNA‐seq revealed that the number of Cluster 0 myocardial isoforms was significantly increased in diabetic myocardium, and the differential genes were mainly enriched in fatty acid metabolism, and the PPARA signaling pathway was found to be over‐activated and involved in the regulation of metabolic reprogramming of diabetic myocardial I/RI.

**Conclusion:**

Metabolic reprogramming of diabetic myocardial I/RI may be the main cause of increased myocardial vulnerability. The number of myocardial subtype Cluster 0 increased significantly, and PPARA PPARA is a ligand‐activated receptor of the nuclear hormone receptor family that plays a central regulatory role in lipid metabolism. signaling pathway activation may be a potential mechanism for reprogramming metabolism in diabetic myocardium.

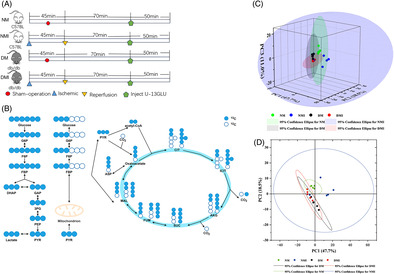

## Graphical abstract

**Highlights**

- Diabetic myocardium undergoes changes in energy metabolism after ischemia–reperfusion injury (I/RI) and impacts myocardial vulnerability.
- Various advanced methods such as mass spectrometry coupled with gas chromatography or liquid chromatography (GC/LC–MS) as well as single‐cell sequencing technology were used.
- Diabetic myocardium undergoes metabolic reprogramming after I/RI, which is mainly characterized by an overdependence on fatty acid metabolism for energy supply.
- Analysis revealed that myocardial subtype Cluster 0 was significantly increased in diabetic myocardium, and the differential gene was mainly enriched in fatty acid metabolism.

## 1 INTRODUCTION

The incidence of diabetes mellitus is rising year by year and has become a worldwide public health problem that seriously threatens human health. Studies have found that the incidence of myocardial ischemia is 1.45–2.99 times higher in diabetic patients than in non‐diabetic patients,^1^ and the incidence of cardiac‐related complications is about 5 times higher than in non‐diabetic patients,^2^ indicating that diabetic myocardial vulnerability is significantly increased, and it is a safety hazard that seriously affects the prognosis and regression of diabetic surgical patients. Ischemia/reperfusion injury (I/RI) is a condition in which tissues or organs undergo a period of ischemia (local tissue hypoxia due to inadequate blood supply), and when blood perfusion is restored, these tissues or organs instead suffer further damage. I/RI is one of the most common factors inducing adverse cardiac events in the perioperative period; therefore, in‐depth exploration of the mechanism of increased susceptibility of diabetic myocardium after the occurrence of I/RI and effective myocardial protection strategies are very important scientific issues.

Currently, there are no clinically targeted preventive and curative measures for the increased susceptibility of diabetic myocardium to I/RI, and most of the basic researches have focused on the exploration of its pathophysiological alterations, including disturbances in energy metabolism, oxidative stress, intracellular calcium overload, apoptosis, autophagy, impaired signaling pathways, and iron death.^3^ Studies have shown that myocardial energy metabolism disorders are the initiating link and important mechanism to induce diabetic myocardial I/RI.^4^, ^5^, ^6^, ^7^ Animal and clinical studies have demonstrated that optimizing energy metabolism, especially promoting glucose metabolism, helps to reduce I/RI and exert cardioprotective effects.^8^, ^9^ However, the specific alterations in metabolic reprogramming of diabetic myocardial I/RI and the underlying mechanisms remain unclear.

The aim of this study was to lay the foundation for seeking effective protective strategies for diabetic myocardium by analyzing the specific alterations in metabolic reprogramming in diabetic mice and healthy mice after they were subjected to I/RI, respectively, and then analyzing the potential mechanisms contributing to metabolic reprogramming at the transcriptomic level of the genes by using single‐cell RNA sequencing (ScRNA‐seq) technology.

## 2 MATERIALS AND METHODS

### 2.1 Experimental animals and experimental modeling

#### 2.1.1 Experimental animals

SPF grade 8–10‐week‐old C57BL male mice and db/db male mice weighing 20–30 g (mice were purchased from Changzhou Cavinston Laboratory Animal Co., Ltd. in Jiangsu Province, China) were used for the study. The use and care of all animals in the experiments were carried out in accordance with the requirements of the European Code of Practice for the Use and Care of Laboratory Animals, and the experiments were approved by the Ethics Committee of the hospital.

#### 2.1.2 I/RI modeling

Experimental mice were anesthetized with sevoflurane. Mice were mechanically ventilated using a small animal ventilator. An incision was made in the third or fourth intercostal space at the left edge of the sternum. The single layer of chest wall muscle was then separated directly to access the chest cavity, and the pericardium was incised to expose the heart. The I/RI group (30 min of ischemia and 120 min of reperfusion) was prepared by ligating the left anterior descending coronary artery (LAD) approximately 2 mm below the root of the left auricle. The sham‐operated group was threaded only without ligation. Mice were executed at 120 min of reperfusion, and myocardial tissue was removed. (Details of the specific experimental steps are given in Data S1).

#### 2.1.3 Experimental groups

Experimental groups include (1) C57BL mouse sham operation group (NM group, *n* = 5), (2) C57BL mouse I/RI group (NMI group, *n* = 5), (3) db/db mouse sham operation group (DM group, *n* = 5), and (4) db/db mouse I/RI group (DMI group, *n* = 5).

### 2.2 Non‐targeted metabolomics

#### 2.2.1 Metabolite extraction

The animal tissue samples were taken, mixed with beads and extraction solution. The extraction solution contains deuterated internal standards. The mixed solution was vortexed for 30 s. Then, the mixed samples were homogenized. The samples were incubate to precipitate proteins. Then, the samples ware centrifuged. The supernatant was transferred to a fresh glass vial for analysis. The quality control (QC) sample was prepared by mixing an equal aliquot of the supernatant of samples. Liquid chromatography–tandem mass spectrometry (LC–MS/MS) analyses were performed using an ultra‐high‐performance liquid chromatography system (Vanquish, Thermo Fisher Scientific) with a Phenomenex Kinetex C18 (2.1 mm × 50 mm, 2.6 μm) coupled to Orbitrap Exploris 120 mass spectrometer (Orbitrap MS, Thermo). (Details of the specific experimental steps are given in Data S1).

### 2.3 Targeted metabolic flow analysis

#### 2.3.1 Experimental animals and modeling

The same animals, modeling procedures, and groupings as in Section 2.1 are used.

#### 2.3.2 U‐13C6‐glucose solution injection procedure

In order to explore more precisely the process of glucose utilization and metabolism in myocardial tissues under different states, and thus indirectly respond to the metabolic status of fatty acids. We use glucose tracking technology to precisely explore the process of glucose utilization and metabolism in myocardial tissues in different states (normal and diabetic conditions), thus indirectly reflecting the metabolic status of fatty acids. For each of the four groups of samples (*n* = 5/group), an in vivo infusion of U‐13C6‐glucose was given as follows (Figure 2A): the sham‐operated groups (NM and DM groups) were injected intraperitoneally with 5% U‐13C‐labeled dextrose (1 mg/g) (purchased from Santa Cruz Biotechnology, Inc., Item No. SC‐239643A, USA) after successful anesthesia, and a caudal IV push of 0.4 mg/g (100 μL) U‐13C glucose, followed by continuous tail vein continuous pumping (at 150 μL/h: 0.012 mg/g/min) for up to 50 min; the I/RI group (NMI and DMI groups) underwent I/RI modeling after successful anesthesia, and then 70 min after reperfusion intraperitoneal injection of 5% U‐13C‐labeled glucose (1 mg/g), 0.4 mg/g (100 μL) of U‐13C glucose was pushed via the tail vein, followed by continuous tail vein continuous pumping (at a rate of 150 μL/h: 0.012 mg/g/min) for up to 50 min, and mouse myocardial tissues were analyzed by targeted metabolomics assay (with assistance from Zhongke Biological Co., Ltd., China).

#### 2.3.3 Sample testing

For tissue samples, extraction solvent was added to equivalent tissue samples and the samples were homogenized. After incubation on ice, the tissue extract was centrifuged. Supernatants were thoroughly lyophilized and reconstituted in methanol water just prior to measurement.

The MS measurement of isotopologue distribution is analyzed via a Thermo QExactive plus hybrid quadrupole–orbitrap mass spectrometer coupled to a Thermo Vanquish UPLC system (Details of the specific experimental steps are given in Data S1).

### 2.4 Experimental methodology of single‐cell nuclear sequencing

#### 2.4.1 Laboratory animals and experimental grouping

Six 8–10‐week‐old male C57BL mice and six male db/db mice were randomly divided into sham‐operated (NM, *n* = 3) and C57 I/RI mice (NMI, *n* = 3); diabetic db/db mice were divided into sham‐operated (DM, *n* = 3) and diabetic db/db I/RI mice (DMI, *n* = 3) respectively (Animals were provided by Changzhou Cavinston Laboratory Animal Co. Ltd. in Jiangsu, China).

#### 2.4.2 Animal modeling and grouping

Same as Section 2.1 (*n* = 3/group).

#### 2.4.3 Experimental procedure and cardiac tissue sampling procedure

The experiments were performed on a 10× Genomics Chromium system utilizing an eight‐channel microfluidic “double cross” system (performed by Shanghai Boho Biotech Co., Ltd., China). The experimental procedure includes single‐cell suspension preparation, single‐cell/nucleus preparation, sorting and library preparation, sequencing, and bioinformatic analysis. (Details of the specific experimental steps are given in Data S1).

## 3 RESULTS

### 3.1 The metabolic reprogramming induced by diabetic myocardial I/RI is mainly manifested as more dependence on fat metabolism for energy, especially the hyperactive metabolism of ultra‐long and medium‐chain fatty acids

Non‐targeted metabolomics allows the identification and relative quantification of multiple metabolites by mass spectrometry coupled with gas chromatography or liquid chromatography (GC/LC–MS). The study was conducted on four groups of samples (NM, NMI, DM, and DMI groups) respectively, and 16 764 peaks were extracted from the experimental samples. In order to reduce the influence of the detection system error on the results and to make the results better highlight the biological significance, a series of preparation and organization of the raw data were carried out. In this project, after the above pre‐processing, finally 11 085 peaks were retained; the details of the number of differential metabolites between groups are shown in Table 1, of which 4466 metabolites with differences, of which 2862 were down‐regulated, and 1604 were down‐regulated.

**TABLE 1:** Summary table of metabolites detected by non‐targeted metabolomics.

Group	Cpd_all	Cpd_diff	Cpd_diff_up	Cpd_diff_down
NM vs. DM	11 085	1447	812	635
NMI vs. DMI	11 085	1133	878	255
NM vs. NMI	11 085	745	318	427
DM vs. DMI	11 085	1141	854	287

Abbreviations: DM, db/db mouse sham operation; DMI, db/db mouse ischemia–reperfusion injury; NM, C57BL mouse sham operation; NMI, C57BL mouse ischemia–reperfusion injury.

**FIGURE 1:**
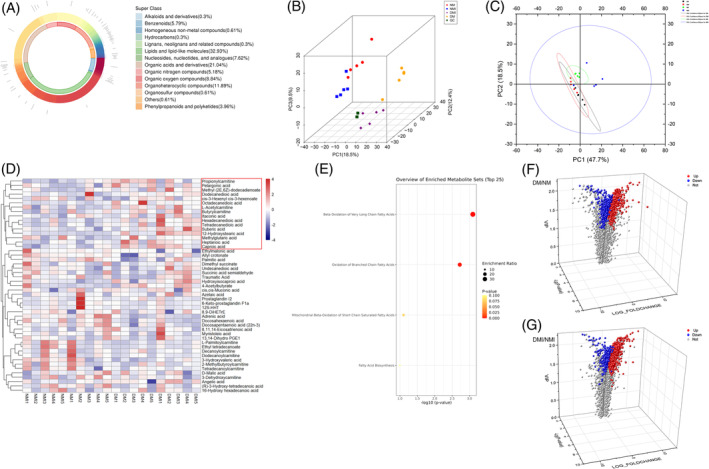
Non‐targeted metabolomic analysis. (A) Metabolite classification and percentage ring plot, different color blocks in the plot indicate different classification categories, and the percentage indicates the metabolites belonging to the type as a percentage of the number of all identified metabolites. (B) Scatterplot of PCA model 3D scores for the four groups. (C) Score scatter plot of OPLS‐DA model for four groups. (D) Heatmap of metabolic differentiators in the DMI group versus the NMI group. (E) KEGG enrichment analysis of differentiated metabolites. (F, G) Differential metabolite volcano plots. DMI, db/db mouse ischemia–reperfusion injury; KEGG, Kyoto Encyclopedia of Genes and Genomes; NMI, C57BL mouse ischemia–reperfusion injury; OPLS‐DA, orthogonal partial least square discriminant analysis; PCA, principal component analysis.

A total of 815 metabolites were detected by GC/LC–MS in the study, categorized, and accounted for alkaloids and their derivatives (0.3%); phenyl‐ring type (5.79%); homogeneous non‐metallic compounds (0.61%); hydrocarbons (0.3%); lignans, neolignans, and related compounds (0.3%); lipids and lipid‐like molecules (32.93%); nucleosides, nucleotides, and analogs (7.62%); organic acids and their derivatives (21.04%); organic nitrogen compounds (5.18%); organic oxygen compounds (8.84%); and organic heterocyclic compounds (11.89%); organosulfur compounds (0.61%); others (0.61%); and phenylpropanoids and polyketides (3.96%) 14 categories; among them, the number of metabolites of lipids and lipid‐like molecules accounted for the highest percentage, which were clustered into six families (Figure 1A). From the proportion of the above categories of metabolites, it is clear that the most important substrates for myocardial energy acquisition are lipids and lipid‐like molecules.

#### 3.1.1 Principal component analysis (PCA) of differential metabolites

PCA is a major method for dimensionality reduction of metabolomic data. PCA of the four groups of heart samples showed that the four clusters were well separated, with high metabolite similarity between NM and NMI, and high metabolite similarity between DM and DMI, but very significant metabolite differences between NM/NMI and DM/DMI (Figure 1B). Particularly by the OPLS‐DA model plot (Figure 1C), it can be significantly found that the metabolite differences between NMI and DMI samples were very significant, The difference in metabolites between NMI and DMI samples was found to be highly significant. A clustered heat map of metabolites (Figure 1D) revealed highly significant differences in metabolites between the NMI and DMI samples, whereas the within‐group differences were not significant, indicating that the metabolic effects of healthy and diabetic myocardium, as well as after being subjected to I/RI, respectively, were relatively obvious, and it is believed that metabolic reprogramming occurred in both cardiomyocytes in order to adapt to the changes in the internal environment.

#### 3.1.2 Cluster analysis of metabolites

After the above preprocessing, the data were normalized, and differences in the accumulation patterns of metabolites in the samples from the four groups (two states and one intervention) were analyzed by drawing clustered heat maps (Figure 1D). The comparative analysis of the difference metabolites in the NM and DM groups, as well as in the NMI and DMI groups, respectively, showed that the differences in metabolites between healthy and diabetic myocardium were highly significant, and the differences in metabolites were even more pronounced after the occurrence of I/RI.

To further analyze the functions of the differential metabolites in the four groups, the analysis revealed that these metabolites were mainly enriched in the oxidation of ultra‐long‐chain fatty acids versus branched‐chain fatty acids by Kyoto Encyclopedia of Genes and Genomes (KEGG) enrichment analysis of these differential metabolites (Figure 1E).

#### 3.1.3 Screening and analysis of differential metabolites

The study screened metabolites from 20 samples and used volcano plots to visualize the differential metabolites screened. The results showed that 812 substances were upregulated in the NM group compared to the DM group; 636 substances were downregulated (Figure 1F); 878 substances were upregulated in the DMI group compared to the NMI group; 255 substances were downregulated (Figure 1G), indicating significant changes in metabolite levels in healthy and diabetic myocardium after exposure to I/RI.

#### 3.1.4 Screening and analysis of differential metabolites of fatty acid and glucose metabolism

In order to more accurately understand the differences in the metabolism of glucose and fatty acids between healthy and diabetic myocardium, the fatty acid and glucose metabolism substrates were screened again, and a total of 48 metabolites were detected (Table 2), and 18 were screened for differential metabolisms; 13 of which were upregulated, and 5 of which were downregulated. It was evident from the metabolite clustering heatmap (Figure S2.1A,B) that the number and expression of fatty acid metabolites were significantly higher in the DM group as well as in the DMI group than in the NM group, indicating that diabetic myocardium relies more on lipid metabolism for energy.

**TABLE 2:** Summary of differential metabolites.

Group	Cpd_all	Cpd_diff	Cpd_diff_up	Cpd_diff_down
DM vs. DMI	48	7	6	1
NM vs. DM	48	3	1	2
NMI vs. DMI	48	6	5	1
NM vs. NMI	48	2	1	1

Abbreviations: DM, db/db mouse sham operation; DMI, db/db mouse ischemia–reperfusion injury; NM, C57BL mouse sham operation; NMI, C57BL mouse ischemia–reperfusion injury.

**FIGURE 2:**
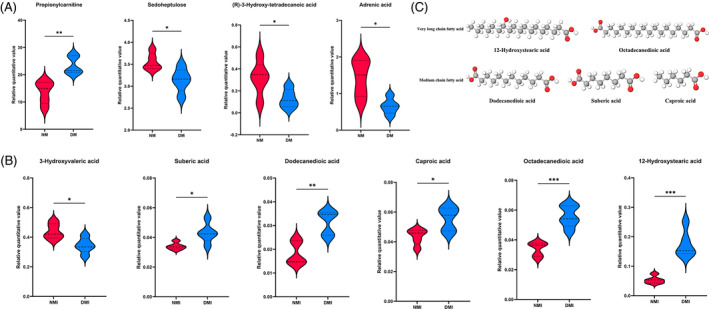
Comparison of differential metabolites between groups and schematic diagram of molecular structure. (A) Differential fatty acid and glucose metabolizers of DM versus NM. (B) Differential fatty acid and glucose metabolizers of DMI versus NMI. (C) Molecular structure diagram. DM, db/db mouse sham operation; DMI, db/db mouse ischemia–reperfusion injury; NM, C57BL mouse sham operation; NMI, C57BL mouse ischemia–reperfusion injury.

A comparison of glucose and fatty acid metabolism differentiators in healthy mice (NM group) and diabetic mice (DM group) revealed a total of four differentiators including (Figure 2A) propionyl carnitine, kingpin heptose, (R)‐3‐hydroxy‐tetradecanoic acid, and adrenoic acid. Propionyl carnitine, also known as C3‐hydroxybutyric acid, is a coenzyme in fat metabolism that can be synthesized by the action of propionyl coenzyme A carboxylase. Propionyl C3‐hydroxybutyrate in the body is usually synthesized from fatty acids and metabolized primarily in the liver, and is an important enzyme in the process of energy metabolism that can be synthesized from fat through the action of propionyl coenzyme A carboxylase. The high expression of this metabolite in diabetic myocardium (DM group) was much higher in healthy myocardium than in the diabetic group, whereas fatty acid metabolism in both groups was characterized by the high expression of the Nestorone sugar; (R)‐3‐hydroxy‐tetradecanoic acid and adrenoic acid products were highly expressed, which may be related to the predominance of cardiomyopathy on fatty acid metabolism energy acquisition. However, its high expression of mesoheptulose in the NM group indicated that glucose utilization in healthy myocardium was higher than that in the diabetic group; for the NMI/DMI comparison (Figure 2B), in addition to the organic acid 3‐hydroxypentanoic acid, which was highly expressed in the NMI, five lipid metabolites (octanedioic acid, dodecanedioic acid, hexanoic acid, 12‐hydroxy stearic acid, and octadecadienoic acid) were highly expressed in the DMI group, and all of these belonged to medium‐ and long‐chain fatty acids, indicating that the myocardium was mainly supplied with energy by fatty acid metabolism, and there was a small portion of glucose in the healthy myocardium, but glucose metabolism of diabetic myocardium was significantly reduced, and diabetic myocardium relied on fatty acid metabolism, especially medium‐ and long‐chain fatty acid metabolism, for energy more obviously after the occurrence of I/RI.

#### 3.1.5 Characterization of differential metabolites

A comparison of NMI vs. DMI revealed that five fatty acid metabolites were highly expressed in the DMI group, 12‐Hydroxystearic acid and octadecanedioic acid belonged to the ultra‐long‐chain fatty acids; dodecanedioic acid, suberic acid, and caproic acid belonged to the medium‐chain fatty acids (Figure 2C). Analysis of the molecular structures of the differential metabolites showed that after the occurrence of I/RI in diabetic myocardium, it is typically characterized by an abnormally active metabolism of mainly ultra‐long and medium‐chain fatty acids.

In summary, by untargeted metabolomics analysis, it was found that myocardium is mainly fed by fatty acids, and metabolic reprogramming occurs after the occurrence of I/RI in both healthy and diabetic myocardium. Compared with healthy myocardium, diabetic myocardium has a significant increase in fatty acid metabolism after I/RI, which is particularly characterized by the abnormally active metabolism of ultra‐long and medium‐chain fatty acids.

### 3.2 Targeted metabolic flow analyses revealed a significant decrease in glucose metabolism after diabetic myocardial I/RI, and an overreliance on fatty acid metabolism as the main manifestation of metabolic reprogramming

**FIGURE 3:**
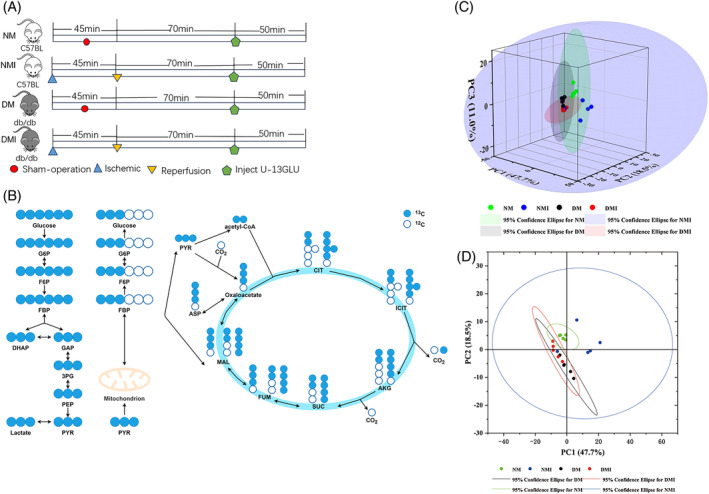
Targeted metabolic flow analysis. (A) Schematic flow of metabolic flow experiment. (B) U‐13C labeled glucose metabolic pathway. (C) Three‐dimensional PCA. (D) Two‐dimensional PCA. PCA, principal component analysis.

To more directly validate the characterization of glucose metabolism in the reprogramming of diabetic myocardial I/RI metabolism, the present study used the isotope U‐13C‐labeled glucose as a tracer for the metabolic flow analysis (Figure 3A,B). A total of 290 differential metabolites were detected in the study, and four groups of samples (*N* = 5) were analyzed separately for comparisons between differential metabolite groups.

#### 3.2.1 Metabolite isotope distribution

Relatively small differences within the four groups were found by PCA, but significant differences in glucose metabolites in cardiac tissues were found among the four groups (Figure 3C,D).

#### 3.2.2 Analysis of differences in the distribution of metabolite isotopes between groups

To study the utilization and metabolism of glucose as a substrate for energy metabolism in myocardial tissues, the following comparative analyses were performed on U‐13C‐labeled glucose uptake, glycolysis, and tricarboxylic acid (TCA) cycling, respectively.

##### U‐ ^13^C‐labeled glucose uptake

**FIGURE 4:**
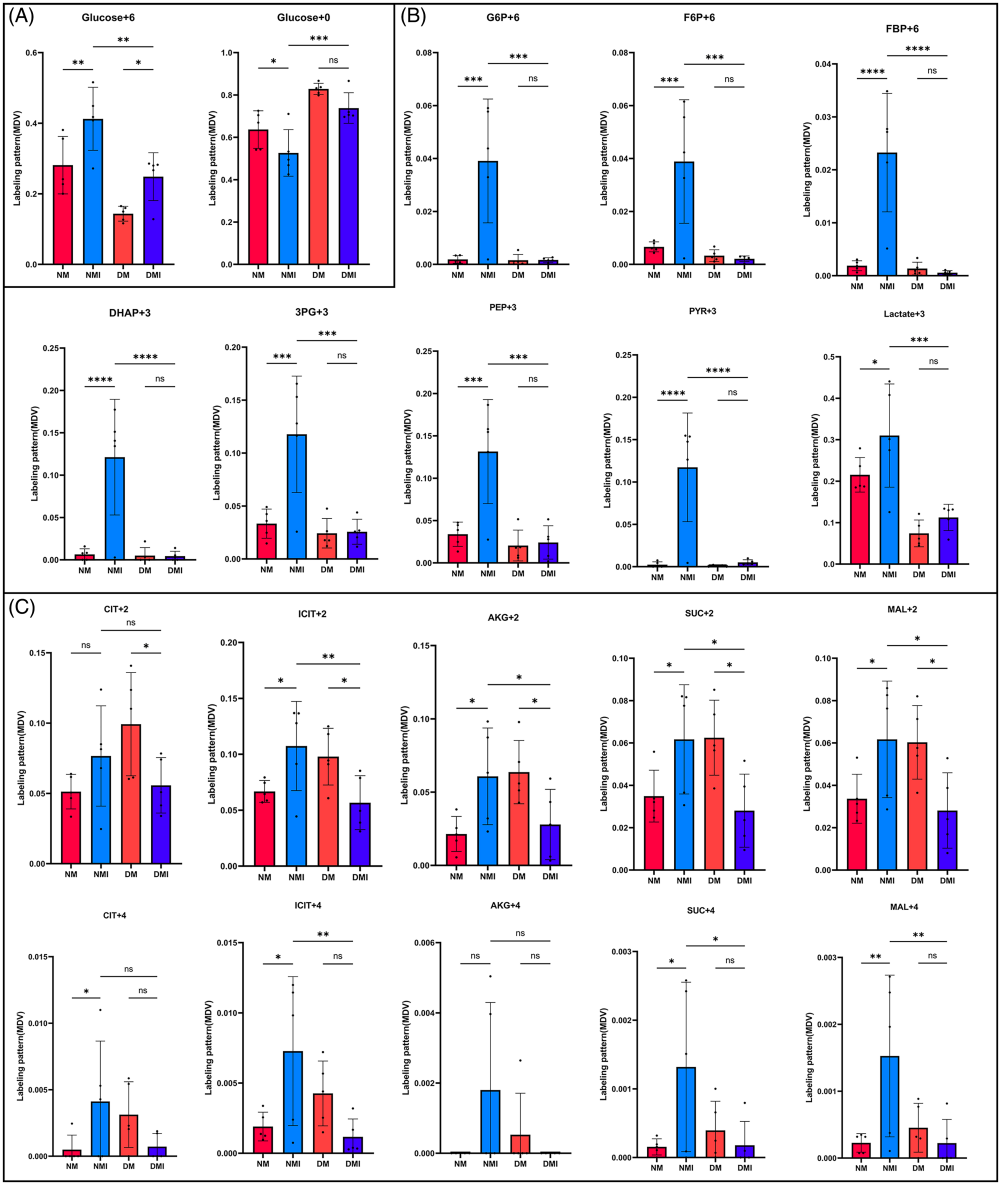
Targeted metabolic flow analysis. (A) Comparison between groups of glucose uptake. (B) Comparison between groups of glycolytic metabolites. (C) Comparison between groups of TTC intermediate metabolites. Variance chi‐square was tested by unpaired *t*‐test; *p* > 0.05 is marked with ns in the graph; *p* < 0.05 is marked with * in the graph; *p* < 0.01 is marked with ** in the graph; *p* < 0.001 is marked with *** in the graph.

A strict fasting preparation of 6 h before the experiment was executed for the four groups of animals, so the level of U‐^13^C‐labeled glucose M + 6 detected during the experiments of the targeted metabolism group could reflect the glucose uptake capacity of the cardiac tissues of the four groups to a certain extent. Analysis of the results of metabolic flow revealed (Figure 4A) that U‐^13^C glucose M + 6 was significantly higher in the NM group than in the DM group, and significantly higher in the NMI group than in the DMI group, and the comparisons were statistically significant (*p* < 0.05), indicating that myocardial uptake of glucose was significantly higher in healthy than in diabetic mice irrespective of whether or not an I/RI had occurred, which is in line with the characteristics of diabetic energy metabolism consistent with the characteristics of diabetic energy metabolism.

U‐^13^C glucose M + 0, which is unlabeled glucose, was found to be significantly higher in the DM group than in the NM group (*p* < 0.05), which, as a side effect, suggests that the myocardial catabolism of glucose as an energy substrate is significantly higher in healthy than in diabetic mice, and it is thought that part of the reason for this is related to the buildup of glucose caused by impaired glucose metabolism in diabetic cardiac muscle.

##### Differential analysis of U‐ ^13^C labeled glucose during glycolysis

Glycolytic pathway metabolites to watch (Figure 4B): Glucose 6‐phosphate (G6P) M + 6, fructose 6‐phosphate (F6P) M + 6, 1,6‐2 fructose phosphate (FBP) M + 6, dihydroxyacetone phosphate (DHAP) M + 3, 3‐phosphoglyceric acid (3PG) M + 3, phosphoenolpyruvate (PEP) M + 3, pyruvate (PYR) M + 3, and lactate (LAC) M + 3 levels. Comparisons of the NM group with the NMI group, the DM group with the DMI group, and the NMI with the DMI group revealed that all of the above metabolites were significantly lower in the NM group than in the NMI group, and the comparisons showed statistically significant differences (*p* < 0.05). In the comparison of the DM with the DMI group, the changes in the above metabolites were not significant, and there was no statistically significant difference (*p* > 0.05). In contrast, in the comparison between NMI and DMI groups, it was found that all of the above metabolites were significantly higher in the NMI group than in the DMI group, and there was a statistically significant difference in the comparison (*p* < 0.05). It suggests that glycolytic processes are significantly enhanced in healthy myocardium after exposure to I/RI, whereas diabetic myocardium is significantly deficient in I/RI glycolysis.

##### Differential analysis of U‐13C‐labeled glucose during the TCA cycle

Glucose metabolism during the TCA cycle requires attention (Figure 4C): citrate (CIT) M + 2/M + 4, isocitrate (ICIT) M + 2/M + 4, a‐ketoglutaric acid (a‐KG) M + 2/M + 4, fumaric acid (fumarate, FUM) M + 2/M + 4, succinate (SUC) M + 2/M + 4, and malate (MAL) M + 2/M + 4. Comparisons of the NM group with the NMI group, the DM group with the DMI group, and the NMI group with the DMI group revealed that the changes in the above metabolites were not significant and there was no statistically significant difference between the NM and the NMI groups (*p* > 0.05); some metabolites in the DMI group were significantly lower than those in the DM group when compared to the DM group, and there was a significant statistically significant difference between the two groups when compared to the DMI group (*p* < 0.05); some metabolites in the NMI group were significantly higher than those in the DMI group, and there was a statistically significant difference in the comparison (*p* < 0.05) The findings suggest that the contribution of glucose as a substrate in the participation of diabetic myocardial I/RI in the TCA cycle is significantly reduced compared to healthy myocardium.

In conclusion, metabolic flow analysis of glucose by isotope U‐13C labeling revealed that the ability of healthy mice to utilize glucose as an energy substrate was significantly higher than that of diabetic mice, glycolytic metabolism was significantly increased in healthy myocardium after the occurrence of I/RI, whereas there was no significant change in glycolysis in diabetic myocardium after I/RI, and the glycolytic process was significantly higher in healthy myocardium after I/RI than in diabetic myocardium. Similarly, the expression level of glucose metabolites during the TCA cycle was much higher in healthy myocardium than in diabetic myocardium, which led to a greater dependence on fatty acids for energy supply in diabetic myocardium after passing through the Randle cycle.

### 3.3 Single‐cell sequencing analysis

ScRNA‐seq is a research technique that has been developed and utilized in genetics in recent years, enabling more precise detection of changes in gene expression at the level of individual cells.^10^, ^11^, ^12^ The study was performed using 10× Genomics' Chromium system utilizing an eight‐channel microfluidic “double cross” crossover system to complete the NM, NMI, DM, and DMI groups (*n* = 3/group). Single‐cell sequencing was performed in the NM, NMI, DM, and DMI groups (*n* = 3/group) on an eight‐channel microfluidic “double‐cross” crossover system (Table 3). A total of 48 320 nuclei were extracted from the four groups of cardiac tissues, and 40 645 nuclei were retained after filtering for QC to create a scRNA‐seq data set.

**TABLE 3:** Number of cells extracted and sequencing gene parameters.

Sample	Actual data	Actual cell number	Median gene number	Mean reads	Total reads
NM	104.65	13 435	1332	25 964	348 826 445
NMI	101.75	9758	1432	34 759	339 182 088
DM	100.17	11 234	1462	29 722	333 902 629
DMI	149.24	13 893	1286	35 805	497 451 830

Abbreviations: DM, db/db mouse sham operation; DMI, db/db mouse ischemia–reperfusion injury; NM, C57BL mouse sham operation; NMI, C57BL mouse ischemia–reperfusion injury.

#### 3.3.1 Cell clustering analysis

**FIGURE 5:**
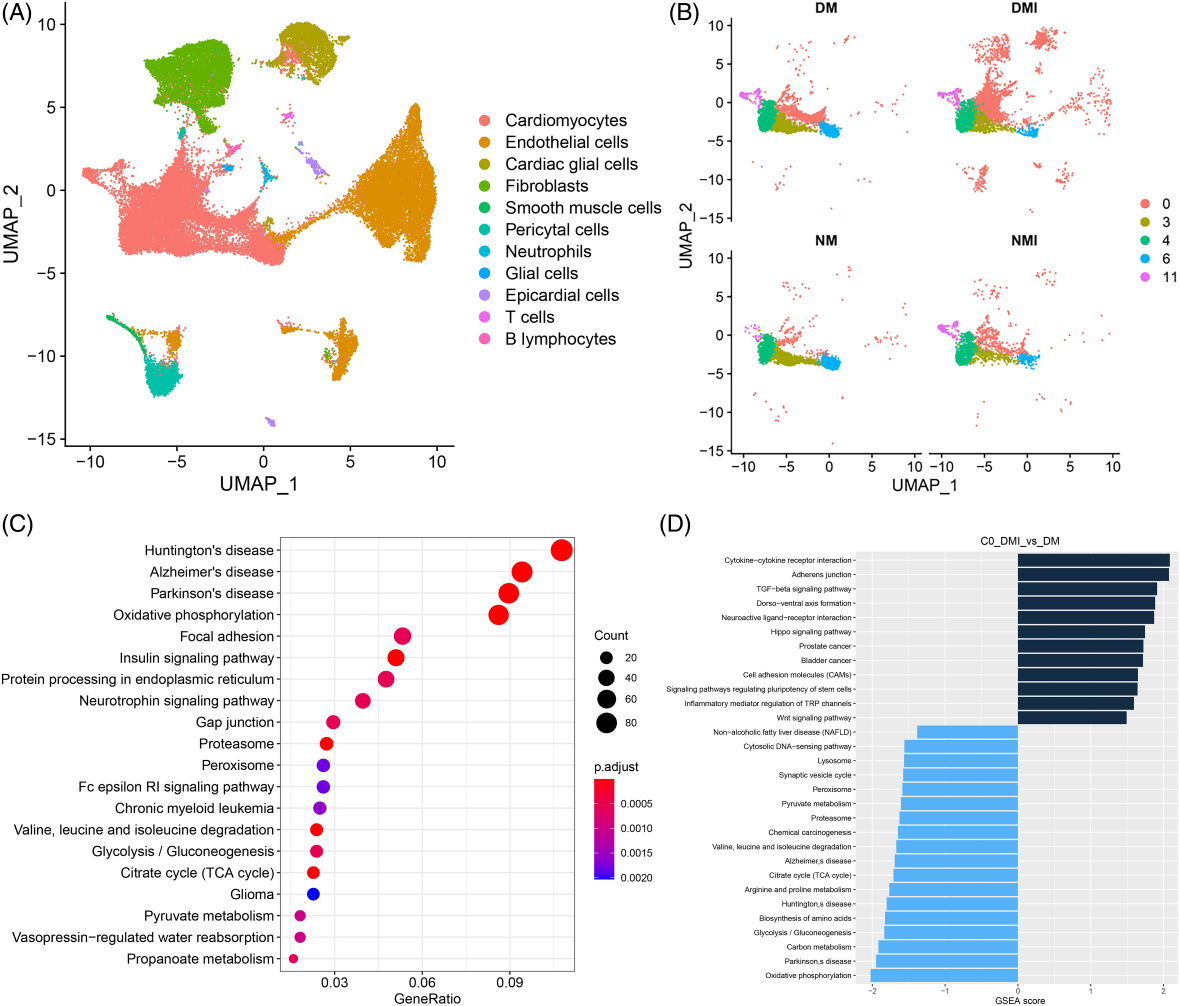
Single‐cell sequencing analysis. (A) UMAP plot showing subpopulation clustering of single‐cell data. (B) UMAP plot showing subpopulation clustering of cardiomyocytes. (C) Bubble plot showing the KEGG analysis results of TOP20 of differential genes between DMI and DM groups. (D) GSEA analysis results of differential genes between DMI and DM groups. DM, db/db mouse sham operation; DMI, db/db mouse ischemia–reperfusion injury; GSEA, gene set enrichment analysis; KEGG, Kyoto Encyclopedia of Genes and Genomes; UMAP, uniform manifold approximation and projection.

Cluster analysis of the sequencing results showed (Figure 5A) that cardiac tissue consisted of 11 cell types, with cardiomyocytes, endothelial cells, and fibroblasts being the most predominant cell types constituting the heart. Cardiomyocytes were categorized into five subpopulations (Figure 5B), among which the Cluster0 subpopulation of cardiomyocytes showed the greatest change in number among the four groups, with a significant increase in the number of Cluster 0 in the DM group compared with the NM group, and a further increase in the number of Cluster 0 in the DMI group compared with the DM group. Therefore, an in‐depth analysis of the abnormal increase in the number share of Cluster 0 was performed to explore the impact on metabolic reprogramming in diabetic myocardium.

#### 3.3.2 Functional analysis of differential genes

A more in‐depth genetic analysis of the Cluster 0 subpopulation of cardiomyocytes was performed, and by gene set enrichment analysis (GSEA) of the differential genes between the DM group and the DMI group of the Cluster 0 subpopulation (Figure 5D), it was found that the functions related to the regulation of the transforming growth factor‐β signaling pathway and inflammatory factors on the transient receptor potential pathway were significantly increased in the DMI group, and the functions related to the TCA cycle, glycolysis/glycolysis, and oxidative phosphorylation were significantly decreased; further analysis by KEGG enrichment (Figure 5C) revealed that the differential genes in the DMI group were mainly enriched to oxidative phosphorylation, glycolysis/glycolysis, TCA cycle, and pyruvate metabolism.

**FIGURE 6:**
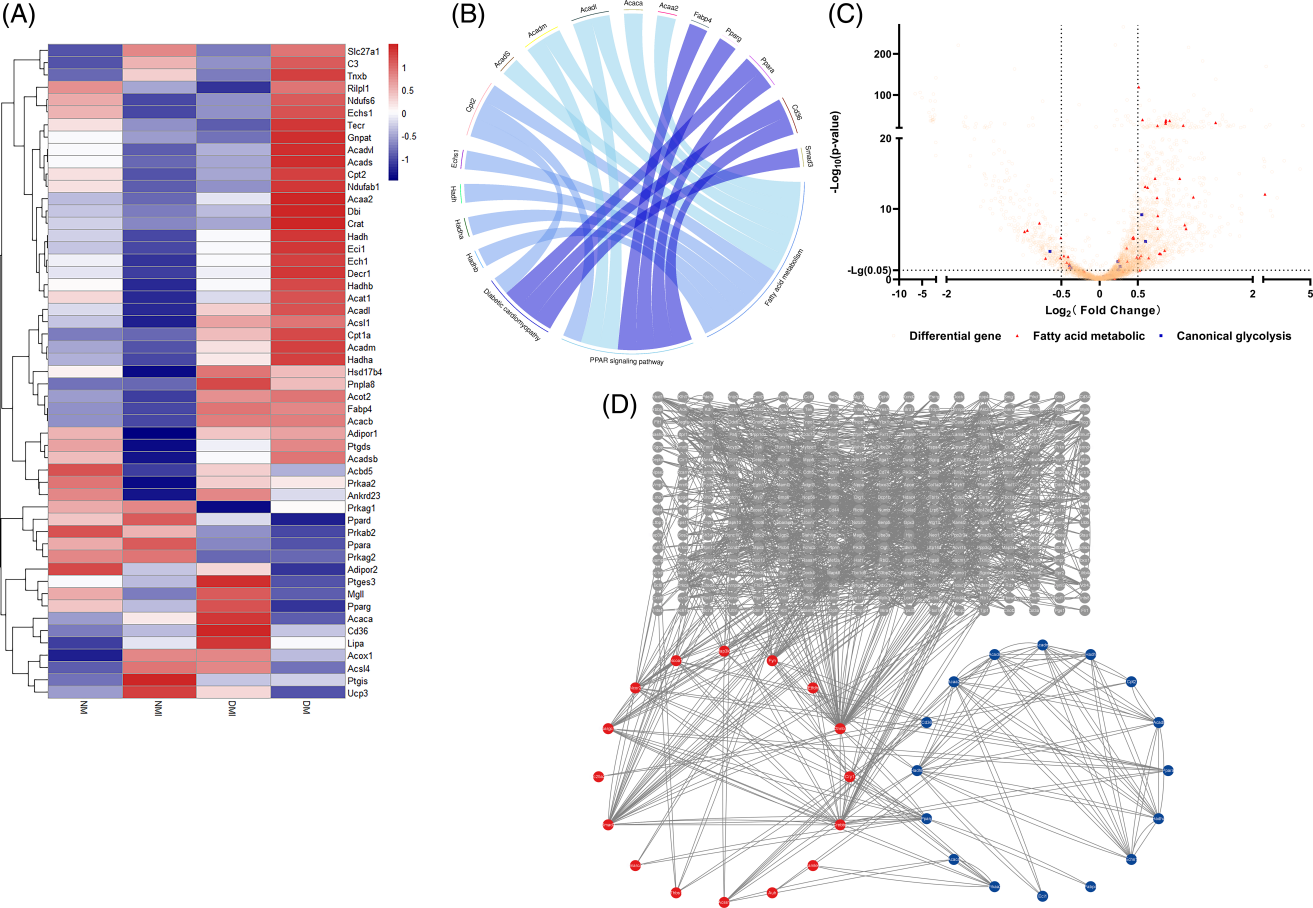
Differential gene analysis and protein interactions analysis plots. (A) Heatmap showing the expression of fatty acid metabolism‐related genes and glucose metabolism‐related genes in DM, NM, DMI and NMI groups. (B) Chordal plot showing the results of GO enrichment analysis. (C) Volcano plot showing the differential genes between DMI and NMI groups (*p*‐value < 0.05, |log2FC| > 0.5). (D) Differential genes of the DMI group of the NMI group of the protein interaction network map (minimum interaction requirement score: 0.900). DM, db/db mouse sham operation; DMI, db/db mouse ischemia–reperfusion injury; GO, gene ontology; NM, C57BL mouse sham operation; NMI, C57BL mouse ischemia–reperfusion injury.

Gene ontology (GO) analysis of the differential genes of the Cluster 0 subpopulations NMI and DMI again revealed (Figure S2.2) that Por, Acadm, Decr1, Dgat1, Cpt2, Eci1, Acadl, Pparg, Akt1, Hadhb, Ppargc1a, Acads, Hadha, Etfdh, Phyh, Adipor1, Acaa2, Hsd17b4, Auh, and Hadh were enriched for fatty acid metabolism and fatty acid oxidation functions. Further analysis revealed that the expression of diabetes I/RI and glucose metabolism–related genes was downregulated, but the expression of fatty acid‐related genes was higher in the DMI group than in the NMI group, and the results should confirm the overdependence on fatty acid metabolism in the DMI group (Figure 6C).

#### 3.3.3 Protein interaction analysis

Differential genes of NMI and DMI were analyzed by protein interaction network (PPI) (Figue 6A), and clear interactions between genes related to fatty acid metabolism were found (Figure 6D). Genes such as Ctnnb of the WNT signaling pathway, MAPK, Map3k7 of the signaling pathway, and the calcium‐regulated protein Camkk2 also interacted with the genes related to fatty acid metabolism, while the genes related to metabolism of Ftfdh, Fyn, and Acss1 metabolism–related genes also interacted with genes related to fatty acid metabolism, and genes such as Ncoa1, Ncor2, Ppargc1a, Smad3, Auh, Crebbp, and Cry1 may be involved in the transcriptional regulation of genes related to fatty acid metabolism. And combined with GO enrichment analysis (Figure 6B), the PPARA signaling pathway was found to be aberrantly activated and involved in metabolic reprogramming of diabetic myocardial I/RI.

In conclusion, the results of scRNA‐seq analysis showed that the number of Cluster 0 myocardial subtypes was significantly increased in diabetic myocardium, and the genetic analysis revealed hyperactivation of the PPARA signaling pathway as a potential mechanism involved in the regulation of reprogramming of I/RI metabolism in diabetic myocardium.

## 4 DISCUSSION

In this study, we used a metabolomic combined with gene transcriptomics approach to explore the potential mechanisms of metabolic reprogramming caused by I/RI in diabetic myocardium by studying metabolite reprogramming after I/RI in healthy and diabetic myocardium, in order to lay a theoretical foundation for the improvement of vulnerability. First, the results of untargeted metabolomics showed that lipid and lipid‐like molecular metabolites are the most important substrates for myocardial energy acquisition. Compared with healthy myocardium, diabetic myocardium after I/RI fatty acid metabolites significantly high expression, especially the significant increase in the metabolism of ultra‐long and medium‐long chain fatty acids as a significant feature, indicating that the diabetic myocardium more dependent on lipid metabolism for energy; again, the isotope U‐13C glucose was used as a tracer for the metabolic flow detection and analysis, and the results showed that compared with the healthy mice, the diabetic mice to the uptake of glucose were significantly reduced and diabetic myocardial I/RI glycolysis was significantly deficient, and the contribution of glucose metabolites during the TCA cycle was significantly reduced, leading to excessive dependence of diabetic myocardium on fatty acids for energy after passing through the Randle cycle. On the basis of the metabolomics results validated using scRNA‐seq analysis, the number of Cluster 0 myocardial isoforms was found to be significantly increased in diabetic myocardium, and the genetic analysis identified overactivation of the PPARA signaling pathway as a potential mechanism regulating the reprogramming of diabetic myocardial I/RI metabolism.

Cellular metabolic reprogramming is a mechanism by which cells promote cell proliferation and growth by changing their metabolic patterns in order to satisfy their energy needs, which not only helps cells to resist external challenges but also confers new functions on them. As early as 2004, Van Bilsen proposed the concept of cardiac metabolic reprogramming.^13^ In the present study, metabolic reprogramming of diabetic myocardium was verified by metabolomics, which was mainly manifested as an overdependence on fatty acid metabolism after I/RI, especially a significant increase in the metabolism of ultra‐long and medium‐long chain fatty acids, which may be an important cause of the significant increase in diabetic myocardial vulnerability.

Under physiological conditions, approximately 70% of the energy of a healthy heart is produced from fatty acids, with the remainder provided mainly by glucose.^14^ Substrates have different energy efficiencies (assessed as the amount of ATP produced by consuming the same amount of oxygen, i.e., P/O ratio). Fatty acid oxidation requires more oxygen to produce more ATP with a P/O ratio of 2.30, while glucose is the most energy efficient substrate with a P/O ratio of 2.58.^15^ In other words for every 1 mole of oxygen consumed, cardiac glucose produces 53.7% more energy than fatty acids from complete oxidation.^16^ Therefore, fatty acids are less oxygen efficient for ATP synthesis compared to glucose. Therefore, increased fat metabolism is an added burden for the myocardium suffering from I/RI.

Cardiomyocytes possess a high degree of flexibility in the selection of substrates for energy metabolism, an energy metabolism trait that appears to be critical for meeting the high energy demands of the myocardium.^17^ Diabetic cardiomyopathy differs from atherosclerotic vascular disease.^18^, ^19^ Its etiology is complex with altered cardiomyocyte metabolism, which is considered a key factor in cardiomyopathic dysfunction.^20^ Diabetes alters myocardial substrate utilization,^21^, ^22^ which leads to decreased glucose oxidation, increased fatty acid oxidation, and decreased glycolysis. This is consistent with our findings.

Although cardiometabolic switching is thought to be an adaptive response to cardiac substrate availability, emerging evidence supports the idea that changes or switches in myocardial substrate use may play an important role in cardiac dysfunction in disease.^23^, ^24^, ^25^ Studies have shown that stimulation of glucose uptake has a consistent protective effect against I/R injury and that stimulation of glucose uptake also contributes to a variety of widely accepted cardioprotective methods, including ischemic preconditioning and insulin treatment.^26^, ^27^, ^28^, ^29^

Disorders of fatty acid metabolism are very harmful to the diabetic myocardium and often lead to cardiac pumping dysfunction. Overdependence on fatty acid metabolism can also cause cardiomyocyte steatosis and hypertrophy due to lipotoxicity, as well as lipid deposition. In addition, increased fatty acid oxidation can lead to an increased rate of oxygen consumption, causing excessive production of reactive oxygen species and reactive nitrogen, endoplasmic reticulum oxidative stress, and mitochondrial uncoupling.^30^ In addition, metabolic reprogramming in diabetic myocardium may also increase the expression of genes related to fatty acid β‐oxidation and inhibit glucose utilization.^31^ In the present study, we found a significant increase in metabolic reprogramming after I/RI in diabetic myocardium, especially in the metabolism of ultra‐long and medium‐long chain fatty acids.

Fatty acid metabolism in organisms is mainly oxidized for energy in mitochondria and peroxisomes, which is an organelle present in almost all eukaryotic cells and plays an important role in many biological processes related to lipid metabolism. Medium‐ and long‐chain fatty acids are mainly oxidized in mitochondria, with only a small fraction oxidized in peroxisomes; whereas ultra‐long‐chain fatty acids are almost exclusively metabolized by β‐oxidation in peroxisomes.^32^ In the present study, we found that reprogramming of diabetic myocardial I/RI metabolism, as manifested by abnormally active fatty acid metabolism in mitochondria and peroxisomes, may be an important link contributing to the increased susceptibility of diabetic myocardial I/RI.

The peroxisome proliferator–activated receptor (PPAR) has been identified to include three isoforms, PPARα (NR1C1), PPARδ/β (NR1C2), and PPARγ (NR1C3),^33^ which belongs to the nuclear receptor (NR) superfamily, and is a ligand‐activated transcription factor that regulates gene expression, and can modulate genes coding for the control of metabolic homeostasis and multiorgan function of protein genes, including the liver, adipose tissue, vascular wall, and heart.^34^ Metabolomic analysis in this study revealed that differential metabolites in diabetic myocardium were mainly in the oxidation of ultra‐long‐chain fatty acids versus branched‐chain fatty acids, and combined with scRNA‐seq analysis of the fatty acid metabolism differential genes and protein interactions in the myocardial C0 subpopulation showed that the PPAR signaling pathway may regulate fatty acid metabolism through Ppara and Pparg, and it is a diabetic myocardial I/RI metabolic reprogramming programming potential mechanism.

In this study, we used a combined analysis of metabolomics and gene transcriptomics to fully validate that metabolic reprogramming occurs in diabetic myocardium after I/RI, which is mainly manifested by an overdependence on fatty acid metabolism for energy acquisition, in particular a significant increase in the metabolism of ultra‐long and medium‐long chain fatty acids, which may be an important contributor to the increase in myocardial susceptibility. ScRNA‐seq revealed that a significant increase in the number of C0 cardiomyocyte subtypes and the PPAR signaling pathway activation by regulating fatty acid metabolism is a potential mechanism for metabolic reprogramming in diabetic myocardium. Subsequently, this theory can be verified by animal experiments, which will open up new ideas for reducing the susceptibility of diabetic myocardium and seeking effective protective measures.

## 5 LIMITATION

The limitations of this study are mainly twofold: first, it has not been empirically validated in animal models by molecular biology experiments. This limitation may have hindered our in‐depth understanding of the causal relationship between the observed metabolic reprogramming phenomena and biological functions. Second, an interventional experimental design has not been involved; therefore, we have not yet been able to determine whether specific pharmacological interventions, such as inhibitors targeting PPARα, are effective in reversing adverse metabolic reprogramming and to assess their potential therapeutic benefits.

## AUTHOR CONTRIBUTIONS

Haping Ma: writing—original draft, validation, visualization, conceptualization, data curation, methodology, project administration, funding acquisition; Jiyao Zhao: formal analysis, methodology, validation, visualization; Yan Zheng: visualization, investigation, data curation, writing—review and editing; Junjie Wang: investigation, writing—review and editing; Yultuz Anwar: investigation; Yuxuan He: writing—review and editing; Jiang Wang: funding acquisition, writing—review and editing.

## CONFLICT OF INTEREST STATEMENT

The authors declare no conflicts of interest.

## Supporting information


**Data S1.** Supporting Information.

## Data Availability

All data generated or analyzed during this study will be available via contacting the corresponding author.
