# Supplementary material for: Potential mechanisms of metabolic reprogramming induced by ischemia–reperfusion injury in diabetic myocardium
Source: J Diabetes. 2024 Oct 25;16(10):e70018. doi: 10.1111/1753-0407.70018 (PMC11503499; doi:10.1111/1753-0407.70018)
Supplement: Supplementary file 1 — Data S1. Supporting Information. [file JDB-16-e70018-s001.docx]

Supplementary Material

# Supplementary Data

**1．I/RI Molding**

Experimental mice were anesthetized with 1.5% sevoflurane and fixed on a rat table after complete disappearance of eyelid and foot reflexes. Laryngoscopy was performed using a 17# trocar needle for tracheal intubation, and mice were mechanically ventilated using a small animal ventilator. Tidal volume was 30-50 ml/kg, respiratory rate was 60 breaths/min, inspiratory-expiratory ratio = 1:2, and FiO2 was 99%. Anal temperature was maintained at 37°C with an animal insulation blanket, and heart rate was continuously monitored using a biosignal acquisition and processing system. An incision was made in the 3rd or 4th intercostal space at the left edge of the sternum. The single layer of chest wall muscle was then separated directly to access the chest cavity, and the pericardium was incised to expose the heart.The I/RI group (30 minutes of ischemia and 120 minutes of reperfusion) was prepared by ligating the left anterior descending coronary artery (LAD) with a 6/0 filament approximately 2 mm below the root of the left auricle. Myocardial ischemia was confirmed by localized epicardial cyanosis below the level of ligation and ST-segment elevation on the electrocardiogram; the sham-operated group was threaded only without ligation. Mice were executed at 120 min of reperfusion, and myocardial tissue was removed.

**2．Non-targeted metabolomics**

2.1.Metabolites Extraction

The animal tissue samples (25 mg±1 mg) were taken, mixed with beads and 500 μL of extraction solution (MeOH:ACN:H2O, 2:2:1 (v/v)). The extraction solution contain deuterated internal standards. The mixed solution were vortexed for 30 s.

Then the mixed samples were homogenized (35 Hz,4 min) and sonicated for 5 min in 4 ℃ water bath, the step repeat for three times.

The samples were incubated for 1 h at -40 ℃ to precipitate proteins. Then the samples ware centrifuged at 12000 rpm (RCF=13800(×g),R= 8.6cm) for 15 min at 4 ℃. The supernatant was transferred to a fresh glass vial for analysis. The quality control (QC) sample was prepared by mixing an equal aliquot of the supernatant of samples.

2.2 LC-MS/MS Analysis

LC-MS/MS analyses were performed using an UHPLC system (Vanquish, Thermo Fisher Scientific) with a Phenomenex Kinetex C18 (2.1 mm × 50 mm, 2.6 μm) coupled to Orbitrap Exploris 120 mass spectrometer (Orbitrap MS, Thermo). The mobile phase A:0.01% acetic acid in water; mobile phase B:IPA:ACN (1:1,v/v). The auto-sampler temperature was 4 ℃, and the injection volume was 2 μL. The Orbitrap Exploris 120 mass spectrometer was used for its ability to acquire MS/MS spectra on information-dependent acquisition (IDA) mode in the control of the acquisition software (Xcalibur, Thermo). In this mode, the acquisition software continuously evaluates the full scan MS spectrum. The ESI source conditions were set as following: sheath gas flow rate as 50 Arb, Aux gas flow rate as 15 Arb, capillary temperature 320 ℃, full MS resolution as 60000, MS/MS resolution as 15000, collision energy: SNCE 20/30/40, spray voltage as 3.8 kV (positive) or -3.4 kV (negative), respectively.

2.3.Data preprocessing and annotation

The raw data were converted to the mzXML format using ProteoWizard and processed with an in-house program, which was developed using R and based on XCMS, for peak detection, extraction, alignment, and integration. Then an in-house MS2 database (Biotree DB) was applied in metabolite annotation. The cutoff for annotation was set at 0.3.

**3．Targeted metabolic flow analysis**

3.1. Experimental animals and modeling: The same animals, modeling process and grouping as in 1.1 are used.

3.2. U-13C6-glucose solution injection process

In order to more accurately explore the myocardial tissue in different states of glucose utilization and metabolism process, so as to indirectly reflect the metabolic status of fatty acids. The 4 groups of samples (n=5/ group) were given U-13C6-glucose infusion in vivo, and the specific procedure was as follows (see Figure 6A) : In the sham operation group (NM and DM groups), 5% U-13C-labeled glucose (1mg/g) was injected intrabitoneal after successful anesthesia (purchased from Santa Cruz Biotechnology Company, article number: SC-239643A, USA), and injected 0.4 mg/g (100 μL) U-13C glucose into the caudal vein, followed by continuous caudal vein pumping (at 150 μL/hr: 0.012 mg/g/min) for up to 50 minutes; The I/RI group (NMI and DMI groups) underwent I/RI modeling after successful anesthesia, and 70 minutes after reperfusion, 5% U-13C-labeled glucose (1mg/g) was intraperitoneally injected, and 0.4 mg/g (100 μL) U-13C glucose was pushed through the tail vein, and then continuous pumping through the tail vein (at a rate of 150 μL/hr) was performed. 0.012 mg/g/min) for up to 50 minutes, mouse myocardial tissue was subjected to targeted metabolomics detection analysis (assisted by Zhongke Biological Co., LTD., China).

**3.2. Sample testing**

For cells and microorganism samples,500 μL cold extraction buffer (methanol: acetonitrile: water = 2:2:1, v/v/v) was added to each sample. Samples were sonicated for 2 minutes and centrifuged at 14,000 g for 5 min at 4 °C. Supernatants were thoroughly lyophilized (FreeZone 6 Liter, Labconco, USA) and reconstituted in 50 μL of methanol-water (1:1, v/v) just prior to measurement.

For tissue samples, 500 μL cold extraction solvent (methanol: acetonitrile: water =2:2:1, v/v/v) was added to equivalent tissue samples and the samples were homogenized at 4°C. After incubation on ice for an additional 20 min, the tissue extract was centrifuged at a speed of 14,000 g for 10 minutes at 4°C. Supernatants were thoroughly lyophilized (FreeZone 6 Liter, Labconco, USA) and reconstituted in 50 μL of methanolwater (1:1, v/v) just prior to measurement.

The MS measurement of isotopologue distribution is analyzed via a Thermo QExactive plus hybrid quadrupole–orbitrap mass spectrometer coupled to a Thermo Vanquish UPLC system. The instrument performance optimization and routine maintenance were performed every 48 h.

3.3. Data analysis

3.3.1. SoftwareData

processing and ion annotation based on accurate mass were performed in TraceFinder 5.0 (Thermo Fisher) and Xcalibur 4.0 (Thermo Fisher).

3.3.2. Calculation of mass distribution vector

Metabolite mass isotopomer distribution (MID, they are also called mass distribution vector, MDV) was determined based on the ratio of the integrated peak areas of the chosen isotopomer to the sum of all the integrated peak areas of the possible isotopomers for the given metabolites.

1.3.3.3. Natural isotope peak correction

Correction for natural abundance is required prior to meaningful interpretation. The correction used both general applicable correction matrix based on Eqn. (1) and isotopic correction matrix that was generated by treating a control group of unlabeled sample.

$$\left( \begin{matrix} I_{0} \\ I_{1} \\ I_{2} \\ \cdots\\ I_{n} \\ \cdots\\ I_{n+u} \end{matrix} \right)=\left( \begin{matrix} L_{0}^{M_{0}} & 0 & 0 & \cdots& 0 \\ L_{1}^{M_{0}} & L_{0}^{M_{1}} & 0 & \cdots& 0 \\ L_{2}^{M_{0}} & L_{1}^{M_{1}} & L_{0}^{M_{2}} & \cdots& 0 \\ \cdots& \cdots& \cdots& \cdots& \cdots\\ L_{n}^{M_{0}} & L_{n-1}^{M_{1}} & L_{n-2}^{M_{2}} & \cdots& \cdots\\ \cdots& \cdots& \cdots& \cdots& \cdots\\ L_{n+u}^{M_{0}} & L_{n+u-1}^{M_{1}} & L_{n+u-2}^{M_{2}} & \cdots& L_{u}^{M_{n}} \end{matrix} \right)\cdot\left( \begin{matrix} M_{0} \\ M_{1} \\ M_{2} \\ \cdots\\ M_{n} \end{matrix} \right)$$

I, the fractional abundances of the measured metabolite ions,

M, the MDV corrected for naturally occurring isotopes,

n, the number of carbon atoms that are present in the analyzed metabolite ion,

u, additional measured ion abundances beyond n originating from natural isotopes in the metabolite,

L, the correction matrix.

**4.Experimental methodology of single-cell nuclear sequencing**

4.1.Laboratory animals and Experimental grouping

six 8-10-week-old male C57BL mice and six male db/db mice were randomly divided into sham-operated (NM，n=3) and C57 I/RI mice (NMI, n=3); diabetic db/db mice were divided into sham-operated (DM, n=3) and diabetic db/db I/RI mice (DMI, n=3)respectively. (animals were provided by Changzhou Cavinston Laboratory Animal Co. Ltd. in Jiangsu, China).

4.2 Single cell sequencing: experimental procedure and cardiac tissue sampling process

The experiment was performed on the Chromium system of 10X Genomics using an 8-channel microfluidic "double cross" cross system (The experiment was performed by Shanghai Bohao Biotechnology Co., LTD., China). The experimental process mainly includes four parts: single cell suspension preparation, single cell/single nucleus preparation, sorting and library preparation, sequencing and bioinformation analysis.

After completion of the maneuvers according to the ischemia-reperfusion modeling or sham procedure, whole hearts were taken after euthanasia for cervical dislocation. Blood was removed from the heart by immediate DPBS perfusion from the mouse aorta; cardiac tissue was collected and placed in liquid nitrogen pre-cooled centrifuge tubes, and the heart tissue was frozen in liquid nitrogen for 1-2 h. Cardiac tissue was pulverized into 1-3-mm pieces, and from each group of 3 heart samples, 10-20 small samples were randomly selected for nucleus extraction. The samples were transferred to cryotubes, snap-frozen in liquid nitrogen, and stored at -80°C until processing Sequencing was performed by Shanghai Boho Biologicals Co. Ltd (Shanghai, China) on a 10xGenomics system (Carlsbad, CA, USA).

4.3. Experimental Flow

4.3.1 10X Genomics onboarding and reverse transcription

(1) The prepared cell suspension, 10X barcode gel magnetic beads and oil were added to different chambers of Chromium Chip G to form GEM (Gel Beads-in-emulsion) via 10X Genomics Chromium system, respectively;

(2) GEM was transferred to a PCR instrument for reverse transcription, and gel magnetic beads containing 30 nt oligo-dT reverse transcription primers allowed poly-A RNA from cells to be reverse transcribed into a single strand of cDNA with Barcode and UMI information;

(3) Magnetic bead purification of one-stranded cDNA;

(4) The purified cDNA was subjected to PCR amplification;

(5) cDNA was assayed for concentration using a Qubit and fragment size using an Agilent 2100.

4.3.2 Sequencing

Cluster generation and hybridization to the first sequencing primer were completed according to the Illumina User Guide, and the flow cell with the cluster was loaded onto the machine. The paired-end program was selected for double-end sequencing. The sequencing process was controlled by Illumina's data collection software and real-time data analysis was performed.

# Supplementary Figure

#
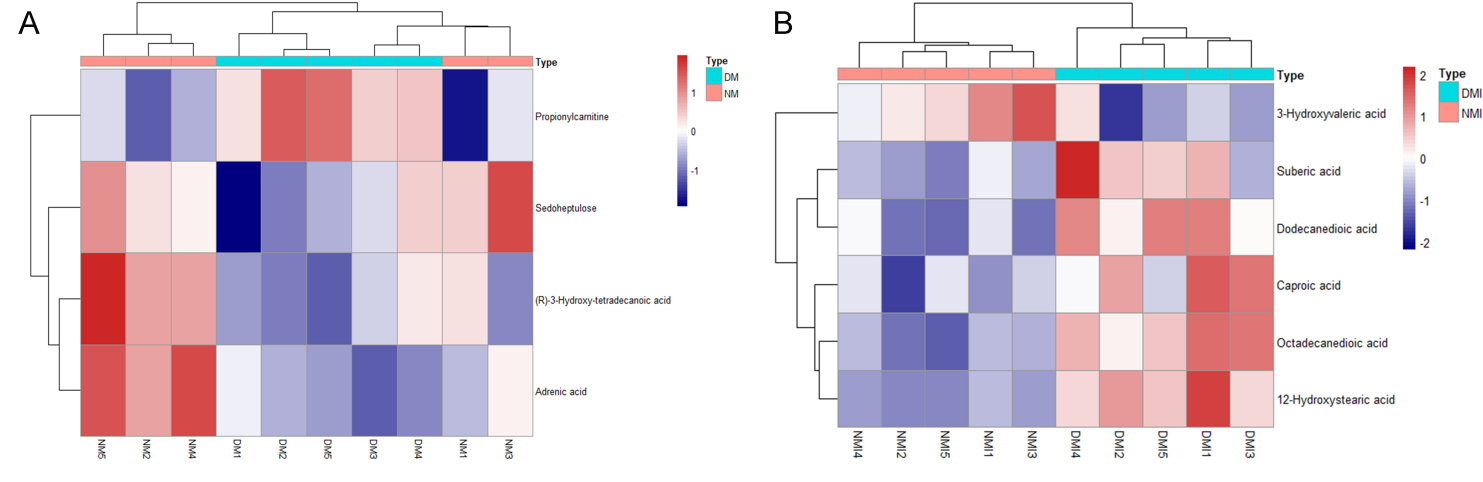


Figure 2.1 Metabolite clustering heat map

Figure Note:A:Differential metabolites of DM VS NM

B:Differential metabolites of DMI VS NMI


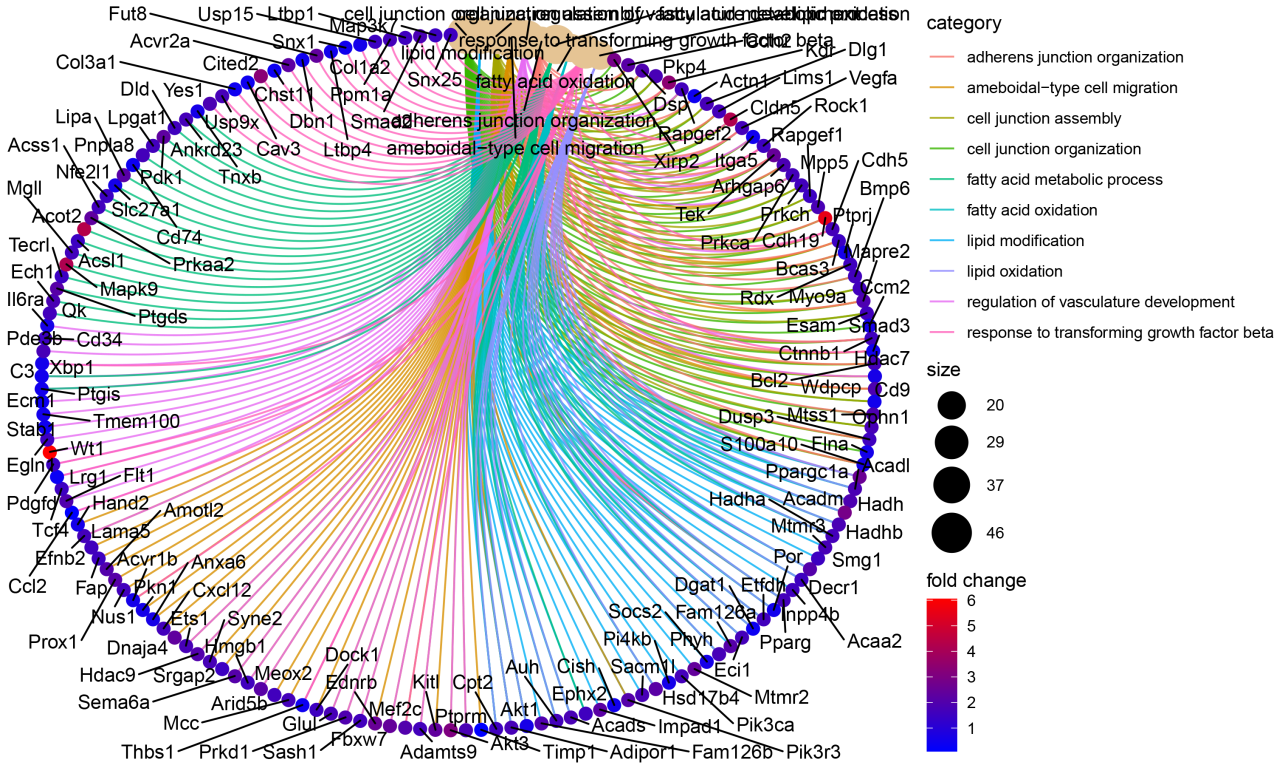


Figure 2.2 GO Analysis Chart

Figure Note:GO analysis of NMI and DMI differential genes in the Cluster0 subpopulation
